# Demystifying Traumatic Experiences and Complex Effects in People with HIV and Post-Traumatic Stress Disorder in Tennessee

**DOI:** 10.1089/heq.2023.0251

**Published:** 2025-02-25

**Authors:** L. Lauren Brown, Almariana J. Acuña, Amna Osman, Lloyda B. Williamson, Carolyn M. Audet, Megan L. Wilkins, Jessica M. Sales, Samantha V. Hill, Jill Foster, April C. Pettit, Latrice C. Pichon

**Affiliations:** ^1^ School of Medicine, Meharry Medical College, Nashville, Tennessee, USA.; ^2^ Nashville CARES, Nashville, Tennessee, USA.; ^3^ School of Medicine, Vanderbilt University Medical Center, Nashville, Tennessee, USA.; ^4^ Department of Health Policy, Vanderbilt University Medical Center, Nashville, Tennessee, USA.; ^5^ St. Jude Children’s Research Hospital, Infectious Diseases, Memphis, Tennessee, USA.; ^6^ School of Public Health, Emory University, Atlanta, Georgia USA.; ^7^ Department of Pediatrics, Emory University, Georgia, USA.; ^8^ University of Minnesota, Minneapolis, Minnesota, USA.; ^9^ School of Public Health, Division of Social and Behavioral Sciences, University of Memphis, Memphis, Tennessee, USA.

**Keywords:** PTSD, HIV, complex trauma, life events, adverse childhood experiences

## Abstract

**Background:**

Compared with the general public, people with HIV (PWH) experience more psychological trauma and higher rates of post-traumatic stress disorder (PTSD), yet limited research explores how PWH may uniquely experience trauma. The primary goal of this study was to investigate trauma exposure typologies and sequelae among PWH to inform trauma screening and interventions.

**Methods:**

Qualitative interviews were conducted with a convenience sample of 20 PWH with PTSD, receiving services from an urban, Tennessee-based HIV Service Organization. Interview guides were conducted to gain a rich understanding of exposure types from the Life Events Checklist-5 (LEC-5), explore potential social determinants of trauma, and uncover effects of chronic trauma or traumata. Thematic content analysis was used to examine typologies and effects.

**Results:**

Exposure typologies appeared as social determinants of trauma, including molestation as the most common followed by racial trauma, community violence, incarceration, addiction, interpersonal violence, poverty cycles, and stigma. Standard PTSD symptoms were reported in addition to emerging effects of complexity, synergism, and resilience. Complex effects spanned socioecological contexts and included sequelae of affective dysregulation, negative self-concept/self-organization, and disturbances in relationships.

**Conclusion:**

Many typologies were not well accounted for in the LEC-5, underscoring the potential to miss exposure types and thus treatment indication. Similarly, effects expanded beyond standard PTSD symptoms, suggesting that nuanced treatment needs may also be overlooked. Findings are consistent with literature indicating the need for updated trauma screening and assessment measures to most comprehensively and accurately direct treatment needs.

## Introduction

Psychological trauma is associated with various adverse health outcomes among people with HIV (PWH).^1^ *Trauma* refers to lasting effects resulting from psychologically distressing experiences, with real or perceived threat of death or serious injury^2^ (e.g., motor vehicle accident, assault, interpersonal violence, molestation).^3^ PWH endure additional exposures related to seroconversion (e.g., diagnosis, stigma, perceived threat to life),^4^ and many report HIV diagnosis as the most traumatic of all experiences.^5^ Long thought to be the most symptomatic trauma disorder, post-traumatic stress disorder (PTSD) is characterized by oscillations between avoidant coping and intrusive re-experiences of stimuli, causing neurophysiological dysregulation and negative cognitions/mood.^2^ Complex PTSD (C-PTSD), however, resulting from chronic trauma usually beginning in childhood, is associated with pervasive disturbances in identity, relationships and self-concept, impulse control, affect dysregulation, and emotional numbing.^6–10^ In total, greater trauma exposure leads to more profound and constant effects, requiring more intensive clinical treatment.^11^

Stemming from an interplay of factors, trauma and HIV interact synergistically as a syndemic^12^ contributing to higher rates of PTSD and an increased disease burden for PWH.^4,13–20^ Syndemic conditions overall are associated with lower adherence to HIV medication,^12,20^ with PTSD specifically increasing risk for disease transmission.^20^ Global PTSD rates among PWH are around 28%, compared with 3.9% in general populations, with highest rates among PWH identifying as cisgender females, cisgender males who have sex with males,^18^ and transgender individuals.^21^

Although the HIV-trauma syndemic is well-documented,^12^ there are limitations to the study and measurement of trauma that can prevent appropriate treatment for PWH.^18^ Exposure tools do not adequately include social determinants of trauma (SDOTs), arising from environmental conditions influencing health (e.g., food security, housing stability, access to resources, racial trauma)^22–25^; thus, there is limited understanding of which sociocultural factors influence the development of PTSD among PWH.^19^ This lack of sensitivity and specificity of exposure detection threatens the accuracy of trauma diagnostics among PWH^18^ and increases the risk for missing referrals to needed care.^26^ To our knowledge, no literature explores C-PTSD among PWH. Research is needed to inform the development of contemporaneous trauma measurement tools^8,18^ to enhance detection of traumata (i.e., cumulative trauma effects) among PWH.^4,26^

We sought to better understand traumata among PWH. An enhanced understanding of trauma exposures and effects among PWH could lead to more accurate screening and assessment tools and more robust treatment modalities, with stronger potential to disrupt the HIV-trauma syndemic.^27,28^

## Methods

### Study Population

We conducted a cross-sectional, qualitative study with a purposive sample of PWH who had participated in a parent study in which all participants were offered a resilience-focused intervention.^29^ Inclusion criteria for the current study entailed that participants had a probable PTSD diagnosis (PTSD Checklist 5 [PCL-5],^30^ ranging from 0 to 80, with a cutoff score ≥31). All participants were 18 years or older and actively receiving services from an HIV Service Organization (HSO) in Nashville, Tennessee, between May 2020 and November 2021.

### Study Procedures

A racially diverse clinical team of HSO personnel (three Black and three White), trained as trauma-specialized licensed master social workers, developed a semistructured interview guide (Appendix I) via four 2-h workgroup meetings to: (1) explore open-ended *happened to me* items from the Life Events Checklist 5 (LEC-5)^8,31^ most predictive of PTSD in the parent study; (2) explore SDOTs such as racial trauma and discrimination; and (3) understand effects of traumata along socioecological contexts of health (e.g., physiological, psychological, social/interpersonal, and/or spiritual).^32^

All participants from the parent study, with a probable PTSD diagnosis, were invited to participate during routine HSO appointments. Interviews, lasting 30–60 min, were conducted by phone (due to COVID-19 mandates), with data entered into Excel in real time by interviewers, as a patient-centered strategy for handling sensitive qualitative data.^33–37^ All participants underwent informed consent processes, provided consent, and received $25 Visa gift cards for completing interviews. Ethics approval was obtained from the Vanderbilt University Human Protection Program (protocol #171596).

### Statistical Analysis

Quantitative results were reviewed from the parent study for the LEC-5 and the PCL-5. Q-Q plots were generated to inspect data normalcy, with mean used for normally distributed data and median otherwise. Qualitative analytical methods followed an inductive–deductive approach using thematic content analysis.^38^ An *a priori* sample size of 20 was sought to determine data saturation. Two cycles of initial coding were conducted, allowing themes to emerge *de novo*, with two investigators (L.L.B. and A.J.A.) conducting coding by entering data into Excel.^38,39^ First cycle codes were organized as descriptions of each LEC-5 item explored. Coders discussed the first item together, and then individually coded the remaining items. Codes were reviewed during second cycle coding, with discrepancies discussed and saturation determined. From here, social determinant typologies^22^ and C-PTSD effects emerged as deductive frameworks^7,40^ applied during the third cycle coding. A codebook of themes was created by separating codes into exposures and effects. Data were then reorganized by participant to gain an understanding of clinical presentation. Theme descriptions were then refined until 100% consensus was reached. The codebook was discussed with a third investigator (L.C.P.) before the final fourth cycle coding.

## Results

### Characteristics of the Study Population

Among 20 PWH enrolled, 10 (50%) identified as cisgender male, nine as cisgender female (40%), and one as transgender female (5%); most (80%) cisgender males identified as same-gender loving; most (*n* = 14, 70%) as Black and six (30%) White, ages from 40 to 65 years (median = 52; interquartile range = 42–61). LEC-5 scores were approximately normally distributed, with a mean of nine events (SD = 1.65). Physical assault was most common (*n* = 19, 95%), followed by assault with a weapon (*n* = 18, 90%), sexual assault (*n* = 16, 80%), other unwanted sexual experiences (*n* = 15, 75%), other stressful experiences (*n* = 13, 65%), severe human suffering (*n* = 12, 60%, and serious injury/harm/death you caused to someone else (*n* = 6, 30%). PCL-5 scores were approximately normally distributed, with a mean score of 53.16 (SD = 15.63).

### Identified Exposure Typologies as SDOTs

Trauma typologies emerged as SDOTs, with the most common typology as molestation (i.e., sexual assault in childhood), followed by racial trauma, poverty cycles, stigma, addiction, interpersonal violence, incarceration, and community violence. All (*n* = 20, 100%) participants experienced ≥1 of the emerging SDOT typologies, with a mean of 3.3 (SD = 1.01). Table 1 includes descriptions and examples of typologies.

**Table 1.** Themes and Example Quotes Depicting Social Determinants of Trauma

Theme and description	Quote
*Molestation:* Watershed events initiating lifelong cycles of damaging or impaired relationships	“That was different because I never had justice. If I had […] it would have been easier for me to get on with my life […] so you gotta’ walk around every day knowing what they did with your body. Having to grow up fast. A woman in a little girl’s body. Once you take innocence from somebody you never get that back. […] they confuse sex with love. -Black cisgender female, age 53
*Racial trauma*	
a) *Ubiquitous and perpetual:* Pervasive, endless, lifelong phenomenon, steeped into fabric of society, experienced intrusively, and limiting quality of life	“… as long as I can remember. It’s just a thing that has always been. In jobs—I have a degree, I went to school, I’m not dumb. They would get the job besides me. My skin color has a lot against me. Not only my prison background—also my skin—I can’t get nowhere because of my past. They discriminate against me because of past and because of my color. […] My color, my race, has hindered me from doing a lot of things. It makes you [want to] give up […] it discourages people. Nothing has changed over the years—it’s still the same.” -Black cisgender female, age 55
b) *Perpetrated by authority figures:* By persons through physical assault or abused power (e.g., police officers, employers, court personnel)	“White police also beat up my brother recently. It’s just not safe out there.” […] “I just don’t trust people—kindness can be reflected on you as a bad thing with some people. They can take advantage of it. I have a hard time trusting anyone but especially white people. That is why I try to stay at home. I can’t get racially discriminated against in my own home where it’s just me. It keeps me inside but then I stay inside so much.” -Black cisgender female, age 49
c) *Minimization/intersectionality:* Denial of racism or conceptual conflation with other minority statuses (e.g., gender identity, poverty, felony).	“I think it was a bit of whiteness issues […] this guy didn’t seem to like white people either. It wasn’t an intentional ‘You’re white and I’m gonna’ kick you out.’ It was more like, “You’re a man who dresses like a woman who happens to be white.” It was in conjunction with everything.” -White transgender female, age 42
*Community violence:* Lifelong experiences, causing ongoing stress, impairing sense of safety	“I could have been killed by someone who hasn’t been caught yet for shooting me. I was shot two times in a drive by. I still think about that, because they still don’t know who it is. I took two bullets. […] It’s a real problem because I think about it a lot because I’m paralyzed in my right hand because of it.” -Black cisgender male, age 64
*Incarceration:* Inhumane or unfair treatment by legal system personnel that exacerbated overall health	“I was in jail … [A tornado] went over the jail; the pod was where the tornado went over. Then we didn’t know if anyone is alive or dead because no one told us anything. The electricity was off for days. […] They didn’t give me my medicine for two months: 62 days. So, I ended up with AIDS as a result. […] They never bothered to send paperwork over to [HIV clinic]. Then they sent me for a forced physical […] They still neglected to get me med[s] while I was there. Then they stood there and talked about my case in front of others—a HIPAA violation. I ended up with AIDS. […] I mean you’re killing people … who should not die for non-violent offenses.”—White transgender female, age 42
*Addiction:* Harm caused to others as a result of substance use	“A lot of rapes. A lot of nights going hungry. Choosing the dope over the food. Being so desperate I would sleep with anyone just to get high. Not bathing. […] The desperation of being homeless and an addict on the street. That’s probably the worst experience I’ve had as a human suffering experience.” -Black cisgender female, age 49
*Interpersonal violence:* Repeated cycles from families of origin through adulthood with romantic partners and family	“Always being in relationships where there’s fighting and sometimes there’s stalking. The majority of my relationship have been like this. […] it impacts my health because it puts a toll on my body; you worry about that person. I have a habit of pulling my hair. Sometimes, I have to go somewhere else to sleep.” -Black cisgender female, age 40
*Poverty cycles:* Protracted periods without food, stable housing, or gainful employment, which led to more trauma exposures	“Trying to get an apartment. Trying to get a job, you know. Meeting my basic needs. Trying to keep a roof over my head. That has been so hard—very stressful. Those things are on my mind all the time.” -Black cisgender male, age 53
*Stigma:* Discrimination related to HIV or other status (e.g., felony), connected with disturbances in relationships (e.g., family estrangement/ abandonment, challenges with romantic relationships)	“I should be past that [accepting HIV status], but I’m not. I’m still dealing with it. It’s always thrown up in my face. I’m reminded when I go to the doctor, when they change my meds, all of that. My family did more damage that anything […] Name calling, stigma. It’s hard to get over your family, people that you love, saying those things. I think that’s my biggest issue. I mean sometimes, I lay up in bed and cry. […] I want to be around my family and want to be home but this stuff from the past still bothers me. […] I think as long as I’m breathing it’ll bother me in some way.” -Black cisgender male, age 58

*Childhood molestation* was the primary theme describing *other unwanted sexual experiences* and *severe human suffering*, discussed by more than half (*n* = 11, 55%) of the participants. These experiences were described as watershed events initiating lifelong cycles of damaging relationships (e.g., violence, mistrust, assault during sex work), exacerbated by parents/family or legal systems not intervening. Long-term difficulty trusting others and perceived “unbreakable cycles” of polyvictimization were connected with incest.

… molestation takes away your childhood. I was between 6–8 years old, and the perpetrator was a “friend’ of the family. Additionally, as an adult in relationships, partners can rape you and that doesn’t make it not rape. -White cisgender female, age 40

*Racial trauma* had three subthemes—*ubiquitous and perpetual*, *perpetrated by authority figures*, and *minimization/intersectionality*—between Black (*n* = 10, 71%) and White (*n* = 4, 67%) participants. When White participants described these experiences, examples indicated attention to intersectional minority status (e.g., sexual orientation, HIV, or gender identity).

The area that I live in has been discriminatory. I have never experienced it until now. You can tell they hate me because I’m white. They have looked at me in complete hate. […] I think also me disclosing my HIV factor is a factor. That has been brought in arguments. -White cisgender female, age 40

When Black participants described these experiences, 8/10 (80%) labeled them as “traumatizing,” and 9/10 (90%) identified that recent local or national racial violence had exacerbated mental health symptoms. *Perpetration by authority figures* was connected to lost financial opportunities and long-term impacts on livelihood.

Racism will never go nowhere. It will always be here. […] It is taught by people who raised children. […] The police we used to look up to are now killing us. You can’t deal with them anymore or you may end up in a body bag. -Black cisgender female, age 53

*Community violence* was endorsed by 10 (50%) participants and connected to ongoing stress that impaired one’s sense of safety.

I have seen people get killed before. It’s nothing around here. It’s common, and I’ve been seeing it since I was a child […] I thought it was okay because that’s all I knew. -Black cisgender female, age 40

*Incarceration* was endorsed by seven (35%) participants and connected to multifactorial traumatic experiences, including inhumane or unfair treatment by legal system personnel, and worsened health or missed opportunities.

When my mother was sick, I was not able to go see her in the hospital. Then she died. They denied me going to the funeral. […] It just hurt me so much. I wanted to react and hurt people. […] I can’t even describe it to you. […] I’ve been at the breaking point many times. […] It all just traumatized me. It was affecting my health, my blood pressure, my nerves. -Black cisgender male, age 57

*Addiction* was endorsed by 13 (65%) participants and focused specifically on substance use by one’s self or the impact of family members’ use during developmental ages.

I caused a lot of harm to my family. I was dealing and using drugs. I […] caught a charge I didn’t deserve. That really affected my family. It hurt them to see me do that and spend so much time in prison. People who love and care about me suffered. -Black cisgender male, age 57

*Interpersonal violence* was endorsed by 13 (65%) participants, often as cyclical in families of origin and continuing through adulthood with romantic partners and family.

The physical abuse I witnessed between my father and mother, and now I carry around the sense that a woman should never be hit period, and I don’t hit anybody in an argument, I will always take the hit. -White cisgender male, age 43

*Poverty cycles* were endorsed by five (25%) participants and connected to contagion for further traumatic experiences (e.g., addiction, coupled with other behaviors such as sex work).

Just living a life of being a homeless drug addict on the street can be a stressful experience. I never want to go through that again. That was the worst out of all the things I’ve experienced. Black cisgender female, age 49

*Stigma* was endorsed by four (20%) related to HIV and/or felony status, with the former seen as a primary contributor to loneliness due to family estrangement/abandonment or challenges with romantic relationships.

After gaining the HIV, I still feel traumatized and feel triggered every time I take the medications. -Black cisgender female, age 50

### Identified Trauma Effects

Trauma effects included standard PTSD symptoms (e.g., avoidance, intrusive thoughts) in addition to the three emerging effects as follows: *complex*, *synergistic*, and *resilience*. *Complex* effects spanned socioecological contexts, aligning with C-PTSD sequelae of affective dysregulation, negative self-concept/self-organization, and disturbances in relationships.^6,7^ Table 2 presents these themes with sample quotes.

**Table 2.** Themes and Additional Sample Quotes Depicting Trauma Effects Among PWH

Multidimensional health effects		
Theme	Subtheme	Quote
*Complex effects along socioecological health contexts*		
a) *Psychological:* Depression, anxiety, lowered self-esteem, rage/aggression, irritability, and violent behaviors.	Disturbances in self-concept	“Not wanting to be around people is a big one—not having a relationship, not having friends. I’m quick to cut people out of my life—I don’t trust people. I think that is because of my trauma. I don’t socialize. I go to work and keep to myself. […] sometimes I have a hard time with my family because of my status. […] I think not accepting things has been a problem for me. I think positively a lot but there is also always a negative side. I try not to let the negative side win but they are always there. -Black cisgender male, age 58
	Impulsivity	“I get angry all the time. I get angry for no apparent reason. I can just be sitting outside and break a chair in half. I don’t know why I don’t like people. And I don’t care about life anymore. […] I have felt suicidal so many times. M— [local mental health organization] can’t help me. I have felt suicidal every time I’ve lost people. I have felt suicidal due to my medications. And my mental health medications have not helped.” -Black cisgender female, age 40
	Affect dysregulation	“My anxiety and OCD—that I didn’t realize it was a real thing […] when it would come time for me to leave the house, I couldn’t leave. I would pace back and forth and practically cry. My anxiety would be so bad. Then I wouldn’t show up or go to work, and then I realized it was my anxiety. I didn’t feel like I deserved to have fun with my friends.” -White cisgender male, age 51
	Emotional numbing	“Losing everything I had, getting beat up and almost killed, and all that … yeah. I’ve been traumatized all my life. My life is not good. It makes me worry about the future. It makes me worry. Don’t nobody call or text me or check on me. […] When I got beat up, it really f—ed me up. My health ain’t been good since then. I hurt every day; I’m confused a lot; I can’t remember s—. Everybody wonders why I get mad. I’m trying to stop smoking and stop drinking. But hell, with everything going on, no wonder I drink.” -White cisgender male, age 64
b) *Interpersonal:* Disturbances in relationships related to withdrawal/self-isolation to avoid reminders of trauma	Disturbances in relationships	“I was molested as a child. I cannot have a successful relationship now […] I just can’t have a successful relationship with a man. He could say a word or something that could remind me, and I just run. It wrecked me mentally and physically. I’m 50 years old now—and I still can’t have a relationship. I just don’t trust men anymore…” -Black cisgender female, age 51
c) *Physiological*: Direct and indirect effects on physical health		“It affected my health problems. I had high blood pressure. I was traumatized and in that hell hole [prison] for 33.5 years. I was ready to say, to hell with all of this with taking care of my health. Stop taking my meds and things.” -Black cisgender male, age 57
d) *Spiritual*: Questioning religious faith, contemplating suicide, or finding inner strength through spiritual beliefs		“… you mad at God, you stay mad at God. You constantly ask him, why it had to be me […] molested, raped and no justice. Why you didn’t do nothing? Are you real? Or are you a myth? […] There were a lot of things I went through in life that I couldn’t bear that turned me to drugs, PTSD, […] and it takes months and months to get through it. It’s just a lifetime healing. You constantly looking for the [*exhale*] now I can take a break. If you don’t deal with your low self-esteem you’ll never feel good. All the work has to come from you. It has to begin with you.” -Black cisgender female, age 53
*Synergistic*: Chronic exposures as contagions for subsequent trauma		“I was molested at a young age. […] it was by someone who was very close to me. I was 12 […] Then before that, my uncle did when I was a little girl. […] It was very confusing for me at that age. [Then later], witnessing my brother get killed by my boyfriend. I couldn’t do anything about it—I just watched. It really affected me—I was 16. It affected my way of thinking. I’ve been going through a life of trauma since I was 2 years old. All from people around me, people that I thought loved me. […] Abusive relationships. […] I was living in bad places, not doing the right things, I dealt with a lot, I saw a lot. I was drinking and using drugs. […] I didn’t want to think or feel and did whatever I could not to. I was self-medicating—using. In bad relationships. Getting involved with the police and getting locked up.” -Black cisgender female, age 55
*Resilience:* Uniquely developed insight about the world, from overcoming adversity or managing challenges, and leveraging resources		“… made me more perceptive to talking to people and learning about them. […] It made me strong. People come to me as if I’m a psychologist—what should I do about this, what should I do about that? People come to me asking me what they should do in their relationships. They tell me I ease their mind—it seems to help out. It makes me feel great too—I like being able to help people. I know one thing, like I said, it made me stronger. I like to talk with people and be around people now—and get to know them. I used to avoid people and not get to know them […]. Now I enjoy being around people.” -Black cisgender male, age 65

*Psychological effects* included the following four categories: (1) *Disturbances in self-concept*, such as lowered self-efficacy/sense of devaluation primarily following molestation; (2) *Impulsivity*, presenting as irritability that easily shifts to volatility, impairing relationships, and leading to involvement with legal systems; (3) *Affect dysregulation*, presenting as avolition, depression, dissociative periods, and lability; and (4) *Emotional numbing,* via maladaptive coping (e.g., substance use).

“… It [molestation and adult sexual assault: polyvictimization] makes you feel devalued and that [is] what you are only good for.” -White cisgender female, age 40

*Interpersonal effects* were most commonly connected to childhood molestation, appearing as disturbances in adult relationships (e.g., Intimate Partner Violence [IPV], inability to trust) and withdrawal despite a recognized need for social ties for coping.

I don’t trust people a lot. And I don’t trust myself around them. […] It makes me introverted, sheltered and stay around my house. I don’t do well in public. I don’t do well when case managers change—people change. […] I open myself up, and then I got to shut down and open up again. I don’t like opening up my wounds over and over again. -Black cisgender female, age 49

*Physiological effects* were *direct*—poor psychological health connected to chronic health conditions—and *indirect*—negative health consequences of maladaptive coping, such as substance use, or psychological sequelae, including avolition/“giving up” or losing motivation to care for one’s self (e.g., abstaining from life-saving medications or food/drink, engaging in risky behaviors).

It put a spike on a drinking spree I went on for about 3 years to deal with things. I used the alcohol to ease the anger. So yeah, my physical health suffered during that time. I took dumb risks and chances that I didn’t need to. Worked myself to death and hurt my body in a lot of ways. Not paying attention to my health, and I lost my leg. -Black cisgender male, age 65

*Spiritual effects* were often described by participants as questioning religious faith or contemplating suicide but also as finding resilience and inner strength through a sense of faith or spiritual beliefs that combated negative thoughts.

It affected everything. It affects me feeling like there is a God sometimes. I just don’t know what to believe. -White cisgender male, age 64

*Synergistic effects* were observed with most participants, stemming from chronic exposures as contagions for subsequent trauma. Intergenerational effects were noted, often related to molestation.

It [traumata] affects my life and my kids’ lives. […] I wouldn’t leave the house. I was paranoid […] that someone would try to kill me or hurt me. […] I still don’t let my kid go stay at peoples’ houses. I don’t like big crowds—I can’t be around a whole lot of people. -Black cisgender female, age 40

Finally, *Resilience* was reported by a large portion of participants as developed from overcoming adversity or managing challenges by leveraging resources (e.g., psychological attributes of tenacity, sustaining beliefs from spirituality/faith, or interpersonal resources from HSO staff and family/friends) or through the sharing of lived experiences, which fostered pride and altruism.

People helped me—they saw the good in me. They gave me a chance, and I took that chance. […] hearing these guys talking positively and boosting me up, I took heed. -Black cisgender male, age 57

## Discussion

Study findings contribute to a growing body of knowledge on trauma exposures and effects. Novel contributions include the study setting (i.e., HSOs are less represented in HIV literature),^41^ rich descriptions of SDOTs among PWH, and observed C-PTSD symptomology. Contextualization is provided for findings and recommendations made for future research.

We observed high rates of all exposures and far-reaching, complex, and synergistic effects. Compared with a large general sample of trauma-exposed people,^42^ almost double reported severe human suffering in our study (60% vs. 31.6%) as well as serious harm to someone else (30% vs. 16.9%). About a quarter more reported other unwanted sexual experiences (75% vs. 48.5%) and other stressful experiences (65% vs. 40.4%). While we did not ask about molestation experiences explicitly, more than half of the participants (55%) reported it, far higher than general samples of males (6%), females (16%),^43^ or other PWH with similar sociodemographic makeup (∼37%).^44^ Overall, observed trauma effects were complex and synergistic but also found to foster resilience. Observed lifelong impairments among participants align with literature showing that childhood adversity contributes to numerous comorbidities,^45^ including worse HIV outcomes,^14,20^ maladaptive coping mechanisms, increased odds of further trauma,^26^ and C-PTSD.^46^

These findings have several implications. First, the connection between molestation and adult comorbidities,^26,45^ including HIV,^13,44^ is well-established, but the inclusion of C-PTSD has often been overlooked.^26^ Explicit detection of molestation may enhance HIV prevention and treatment.^27,28^ Second, certain LEC-5 typologies have been associated with different trauma disorders (PTSD vs. C-PTSD) (e.g.,^47^ sexual trauma correlates with emotion dysregulation and suicide attempts, and assault correlates with substance use disorder).^8,15,18,47^ Thus, limits to the scope or accuracy of screening tools could impede the recognition of some exposures or increase the likelihood for inappropriate treatment modalities. Third, the LEC-5 was chosen as a gold standard instrument, but it was not developed to identify experiences that may be unique to PWH^8,15,18,48^ and contains vague language/descriptions (e.g., “severe human suffering”) that should be further validated among PWH. Fourth, our findings on SDOTs support past literature on SDOTs and underscore the utility of integrating SDOTs into screening tools. In summary, results suggest need to refine trauma measurement and diagnostic tools to yield more accurate results to enable more expeditious referrals to tailored treatment.^49–51^

Observed SDOT typologies are supported by literature^15^,^18^,^52–62^ and include molestation, racial trauma, community violence, incarceration, addiction, interpersonal violence, poverty cycles, and stigma. These exposures have not generally been included in other trauma exposure scales^63–65^ and should be explored and validated in future research. Finally, greater efforts should be taken to focus on HSOs as an understudied setting for which PWH may feel comfortable addressing traumata.^29^

## Limitations

Results cannot be generalized beyond the current dataset of purposively sampled PWH and PTSD in the southern United States, with a median age of 52 years, comparatively older than the majority of PWH in the United States. Trauma typologies and effects explored were not exhaustive but instead focused on exploring experiences presenting as relevant to the parent study. Diagnostic assessments were limited to the PCL-5 and PTSD, which precluded some germane sequelae (e.g., dissociation, which is strongly associated with C-PTSD).^8^ Finally, the phone format and *in vivo* note-taking may have led to data loss. Replication of this study is needed in younger samples, in other regions, using broader inclusion criteria, and exploring other sequelae. Future studies should more pointedly explore the various forms of resilience PWH have fostered in light of the overall trauma burden.

## Conclusion

Descriptions of previously undefined trauma exposures and effects were explored among PWH and PTSD in the underresearched HSO setting. Results are consistent with a growing body of literature suggesting a need for updating trauma measurement tools. Findings provide a deeper understanding of how some PWH with PTSD experience complex symptomology, highlighting the potential benefit of trauma-informed HIV care. Future research can build on these findings by exploring the validity of the emerging trauma typologies and incorporating C-PTSD assessment into protocols.

## Abbreviations Used

C-PTSD: Complex post-traumatic stress disorder
HSO: HIV Service Organization
IPV: Intimate partner violence
LEC-5: Life Events Checklist-5
PWH: People with HIV
PCL-5: PTSD Checklist-5
PTSD: Post-traumatic stress disorder
SDOT: Social determinants of trauma

## Appendix

**Table:** Appendix I. Interview Guide

Domain: Unwanted sexual experiences	➣ One of the questions we asked was if you had personally experienced sexual assault or “other unwanted sexual experiences.” the preliminary results of our trauma study suggest experiences of sexual assault in general were not predictors of post-traumatic stress disorder, but “other unwanted sexual experiences were.” We are interested to learn more about what the clients of—may have experienced as unwanted sexual experiences.
Domain: Severe human suffering	We also asked people if they had experienced “severe human suffering” and found that people who said they had experienced “severe human suffering” were 2.5 times more at risk for PTSD than those who had not experienced severe human suffering. If you answered “yes” that you had experienced severe human suffering, could you describe those experiences?
	➣*Could you describe those experiences—how you have suffered and what you think might help you to improve this suffering?*
	➣ Can you describe how you have suffered?
	➣ And what do you think might help you to improve this suffering?
Domain: Harm to someone else	➣ Another question we asked about was “Serious injury/harm/death you caused someone else.” Our preliminary results show people who answered “yes” to this were more than 5 times more likely to meet the diagnosis for PTSD. If you answered yes to this item, would you be comfortable to tell us more about your experience.
Domain: Other stressful life experiences	We asked about many different types of traumatic experiences. You may remember some of the questions. As a reminder, we asked if people had personally experienced a natural disaster, fire, accident at work or home, sexual assault, life-threatening illness or serious injury, motor vehicle accidents, physical assault with and without a weapon, and a few others. Though we covered a lot of different types of experiences, many people said they had “Other stressful life experiences” that were not included in this list, and “other stressful life experiences” were also connected with post-traumatic stress disorder.
	➣Can you think about what “other stressful life experience(s)” you may have had. If so, can you describe some of these “other” types of stressful life experiences?
Domain: Effects of traumata	As part of our trauma program, when we first asked you about your experiences, we asked about many different types of potentially traumatic experiences. After doing statistical testing we found that clients who participated in the program had experienced an average of 5 or more traumatic experiences in their lifetime. We also found that people who had experienced multiple traumatic experiences had an increased risk for PTSD. If you have experienced multiple traumatic life events, could you tell me how those experiences have impacted your life?
	➣Do you think working with our trauma team helped improve any of the difficulties you were experiencing? Did you experience any changes as a result of working with a trauma specialist at CARES?
	Racial/Ethnic Discrimination Item 6: We also found that many people said they had experienced discrimination related to their race or ethnicity. Some of the questions we asked included perceived discrimination at work, school, or in public places with police or security guards. We would like to ask you a few questions about any racial or ethnic discrimination you may have experienced. This may be a difficult topic for many to discuss right now, given what has been happening throughout our country. Please let me know if you would like a break while we are talking. ➣ First, can you tell us, generally, about any discrimination you may have experienced as a result of your race or ethnicity?
	Given that you have gone through our trauma program you probably have a good amount of knowledge about what trauma is and how it sometimes affects someone’s health [you have a strong understanding of what we mean by “trauma”], I would like to ask how any experiences of racial/ethnic discrimination may have been traumatizing for you. Specifically, we would like to better understand how these experiences have impacted your ability to take care of yourself emotionally/psychologically, physically, spiritually, and so forth.
	o Emotionally/psychologically:
	o Physically overall and with HIV:
	o Spiritually:
	Finally, in light of the recent events in the world, in our country, and specifically in our city, I want to ask you how have you been impacted by the recent tornado, COVID-19 pandemic, and racial violence? Specifically, have any of these events impacted your overall health?…starting first with ➣ The tornado: Would you say your health suffered from the aftermath of the Nashville tornado directly or indirectly?
